# DNA Strand Break Properties of Protoporphyrin IX by X-ray Irradiation against Melanoma

**DOI:** 10.3390/ijms21072302

**Published:** 2020-03-26

**Authors:** Takema Hasegawa, Junko Takahashi, Shinsuke Nagasawa, Motomichi Doi, Akihiro Moriyama, Hitoshi Iwahashi

**Affiliations:** 1 The United Graduate School of Agricultural Science, Gifu University, 1-1 Yanagido, Gifu, Gifu 501-1193, Japan; h5takema@gmail.com (T.H.); m.aki729dabcpi@gmail.com (A.M.); h1884@gifu-u.ac.jp (H.I.); 2 Biomedical Research Institute, National Institute of Advanced Industrial Science and Technology (AIST), 1-1-1 Higashi, Tsukuba, Ibaraki 305-8566, Japan; doi-m@aist.go.jp; 3 Department of Radiology, Graduate School of Medical Science, Kyoto Prefectural University of Medicine, 465 Kajii-cho, Kawaramachi-Hirokoji, Kamigyo-ku, Kyoto 602-8566, Japan; snaga@koto.kpu-m.ac.jp; 4 DAILAB, National Institute of Advanced Industrial Science and Technology (AIST), 1-1-1 Higashi, Tsukuba, Ibaraki 305-8566, Japan

**Keywords:** protoporphyrin IX, X-ray, DNA double-strand break, reactive oxygen species (ROS), radiotherapy, melanoma, radiodynamic therapy, photodynamic therapy

## Abstract

Recent reports have suggested that 5-aminolevulinic acid (5-ALA), which is a precursor to protoporphyrin IX (PpIX), leads to selective accumulation of PpIX in tumor cells and acts as a radiation sensitizer in vitro and in vivo in mouse models of melanoma, glioma, and colon cancer. In this study, we investigated the effect of PpIX under X-ray irradiation through ROS generation and DNA damage. ROS generation by the interaction between PpIX and X-ray was evaluated by two kinds of probes, 3′-(p-aminophenyl) fluorescein (APF) for hydroxyl radical (•OH) detection and dihydroethidium (DHE) for superoxide (O_2_^•-^). •OH showed an increase, regardless of the dissolved oxygen. Meanwhile, the increase in O_2_^•-^ was proportional to the dissolved oxygen. Strand breaks (SBs) of DNA molecule were evaluated by gel electrophoresis, and the enhancement of SBs was observed by PpIX treatment. We also studied the effect of PpIX for DNA damage in cells by X-ray irradiation using a B16 melanoma culture. X-ray irradiation induced γH2AX, DNA double-strand breaks (DSBs) in the context of chromatin, and affected cell survival. Since PpIX can enhance ROS generation even in a hypoxic state and induce DNA damage, combined radiotherapy treatment with 5-ALA is expected to improve therapeutic efficacy for radioresistant tumors.

## 1. Introduction

Radiotherapy (RT) is one of the major therapies for cancer [1,2]. The total clinical radiation dose is limited to a threshold value to avoid causing any damage to normal cells [3,4]. To overcome this issue, a series of studies on the combination treatment of 5-aminolevulinic acid (5-ALA) and ionizing radiation have been conducted by some research groups using mouse models of melanoma, glioma, and colon cancer [5,6,7,8,9,10,11,12,13,14,15]. It is known that 5-ALA administration specifically results in the accumulation of protoporphyrin IX (PpIX) in cancer cells by inhibiting the conversion PpIX to heme [16,17]. These studies have shown a radiosensitizing effect of 5-ALA as a precursor to PpIX.

Accumulation to the tumor and subsequent excitation by laser beam causes PpIX to take a singlet state, which emits fluorescence upon returning to the ground state, accompanied by reactive oxygen species (ROS) production. Hence, 5-ALA has been used for photodynamic therapy (PDT) [18,19]. The treatment of PDT utilizing 5-ALA started with skin cancer and is currently being applied to various kinds of cancers. 5-ALA has also gained clinical approval for intraoperative photodynamic diagnosis (PDD) of malignant glioma in many countries [20]. Therefore, the administration of 5-ALA is already accepted. 5-ALA PDT is used to treat Bowen’s disease (BD), but recurrence and tumor cell persistence after 5-ALA-PDT is sometimes problematic. Combination therapy with 5-ALA-PDT and RT for four patients has been evaluated with the expectation that the cure rate of BD could be improved [21].

PpIX has the ability of ROS generation under X-ray irradiation [22]. However, a robust physics-based explanation for the 5-ALA-PpIX radiosensitizing effect is currently lacking [23]. One of the characteristics of PpIX as a photosensitizer is its ability to undergo photobleaching [24,25]. Photobleaching of PpIX occurs through direct degradation by light irradiation [26]. Previously, bleaching of PpIX by X-ray irradiation was reported with a liquid phase [6]. This finding is also evidence, showing that PpIX causes a physical reaction by X-ray irradiation. Verification of the radiosensitizing effect of 5-ALA would be required through the accumulation of such reactions. It has also been reported that the major damage to cancer cells by X-ray stems from ROS and that these ROS are responsible for DNA breaks [27]. In this study, we analyzed the role of ROS generated from PpIX under X-ray irradiation in regard to strand breaks (SBs) for a physics-based explanation. We also studied the ability of PpIX to cause DNA damage in vitro using a B16 melanoma culture.

## 2. Results

### 2.1. PpIX Enhances ROS Generation by X-ray Irradiation

**Figure 1:**
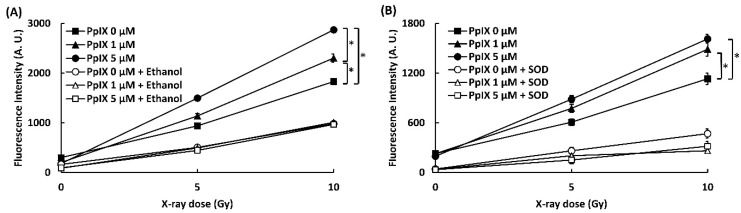
PpIX (protoporphyrin IX) enhanced ROS generation by X-ray irradiation. (**A**) ^•^OH measured by 3′-(p-aminophenyl) fluorescein (APF). Ethanol was used as a scavenger for ^•^OH. (**B**) O_2_^•-^ measured by dihydroethidium (DHE). Superoxide dismutase (SOD) was used as a scavenger for O_2_^•-^. (Data given with *n* = 4, *: *p* < 0.01 in a one-way ANOVA and Tukey post-test)

To confirm that the interaction between PpIX and X-ray leads to the generation of ROS, ROS generations (•OH and O_2_^•-^) were evaluated by two kinds of probes, 3′-(p-aminophenyl) fluorescein (APF) for hydroxyl radical (•OH) detection and dihydroethidium (DHE) for superoxide (O_2_^•-^). The effect of ethanol and DMSO as a quencher of ROS was also evaluated. Figure 1 shows the amounts of ROS generated (^•^OH and O_2_^•-^). The fluorescence intensity of APF and DHE increased in proportion to the concentration of PpIX and the X-ray irradiation dose. Significant differences in ROS generation by X-ray irradiation were found in 0, 1, and 5 µM PpIX solutions. The addition of ethanol and superoxide dismutase (SOD) as scavengers for respective ROS decreased fluorescence intensity of APF and DHE. This occurrence meant that an increase in fluorescence intensity resulted in higher ROS generation. Thus, we confirmed that PpIX enhanced ^•^OH and O_2_^•-^ generation by X-ray irradiation.

### 2.2. Effect of Dissolved Oxygen on ROS Generation by the Interaction between X-ray and PpIX

**Figure 2:**
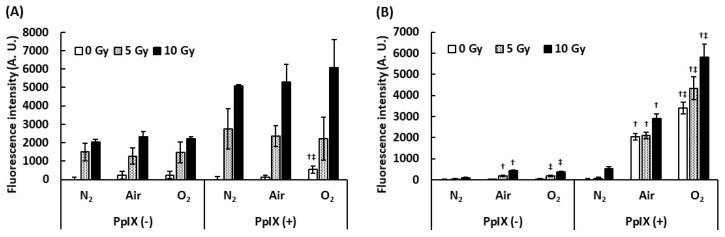
Effect of dissolved oxygen on ROS generation by the interaction between PpIX and X-ray. The PpIX mixture was bubbled by N_2_, air, and O_2_ gas. (**A**) **^•^**OH measured by APF. (**B**) O_2_^•-^ measured by DHE. (Data given with *n* = 6, †: *p* < 0.01 vs. the same X-ray dose (Gy) under the N_2_ gas bubbling condition, ‡: *p* < 0.01 vs. the same X-ray dose (Gy) under the air bubbling condition in a one-way ANOVA and Tukey post-test)

To understand the effect of dissolved oxygen on ROS generation by the interaction between PpIX and X-ray, we measured ROS under different dissolved oxygen conditions. Dissolved oxygen was controlled by bubbling with N_2_ gas, air, and O_2_ gas, and these PpIX solutions were irradiated with X-rays. Figure 2 illustrates the evaluation of the effect of dissolved oxygen on ROS generation. The amount of ^•^OH generated with PpIX was more than that generated without PpIX under each bubbling condition. Even under the N_2_ gas bubbling condition, the amount of ^•^OH generated did not decrease. It was suggested that ^•^OH was derived from H_2_O and not from dissolved oxygen. We found that PpIX enhanced the ^•^OH generation reaction with H_2_O and X-ray regardless of the amount of dissolved oxygen. In contrast, significant differences were found in O_2_^•-^ generation by the interaction between X-ray and PpIX in each bubbling condition. The increase in the amount of O_2_^•-^ generated with PpIX was directly proportional to the amount of dissolved oxygen. O_2_^•-^ was not generated under the N_2_ gas bubbling condition. It was suggested that O_2_^•-^ was derived from the dissolved oxygen, and PpIX enhanced O_2_^•-^ generation under each dissolved oxygen condition.

### 2.3. PpIX Enhances X-ray Irradiation-mediated Single-strand Breaks (SSBs) and Double-strand Breaks (DSBs)

**Figure 3:**
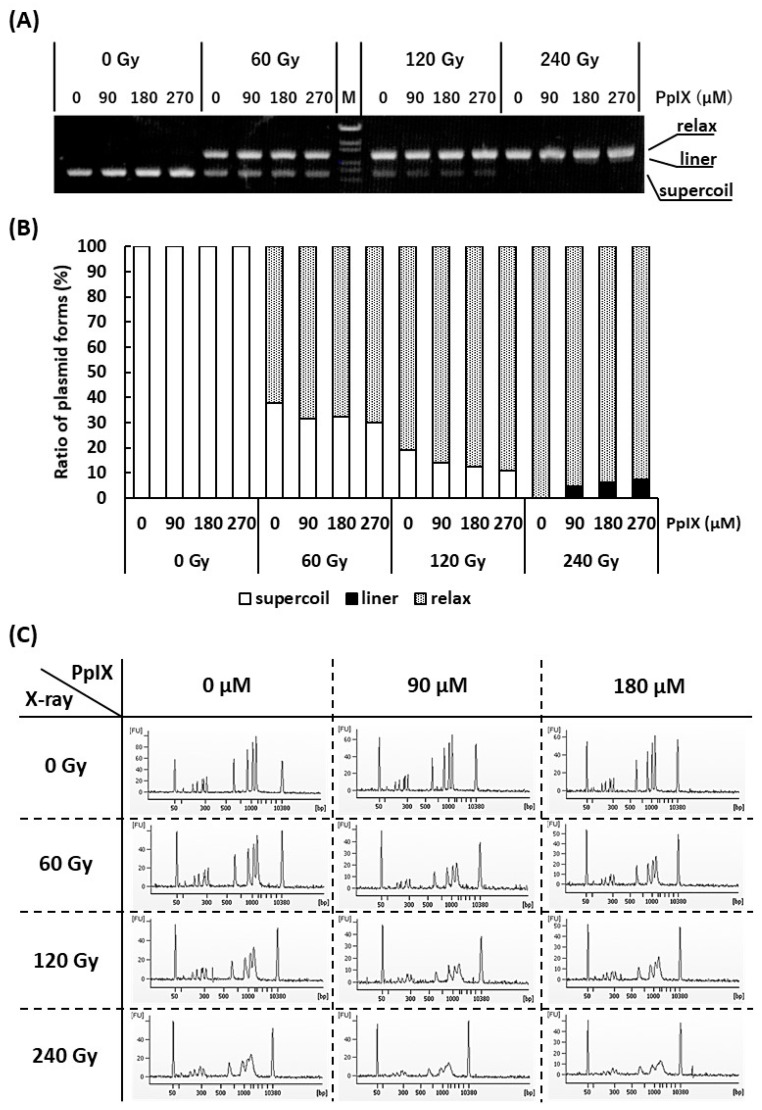
Evaluation of strand breaks (SBs) by X-ray irradiation and enhancement of SBs by PpIX for plasmid pBR322. (**A**) Agarose gel electrophoresis (0.7% agarose) of plasmids irradiated with X-ray at different concentrations of PpIX. “M” is the DNA ladder [OneSTEP Marker 6 (λ/Sty I digest), Nippon Gene, Japan]. (**B**) The fluorescence intensity of (**A**) was quantified, and the ratio of supercoiled to relaxed plasmid was calculated. (**C**) Capillary gel electrophoresis of DNA ladder mixed with PpIX and irradiated with X-ray. The 50 bp and 10,380 bp peaks belong to an internal standard marker.

It is often reported that both X-ray and ROS attack the DNA and cause SBs [28]. Since it has been confirmed that PpIX enhances ROS generation by X-ray irradiation, the generated ROS may enhance SBs. To verify this hypothesis, plasmid (pBR322), as a kind of DNA molecule, and different concentrations of PpIX were mixed and irradiated with X-ray. Figure 3A,B show agarose gel electrophoresis and the ratios of supercoiled, relaxed, and linear plasmid forms, respectively. Plasmids are usually supercoiled, but if SSBs occur, the plasmid form changes to relaxed. Thus, X-ray irradiation, enhanced by PpIX, induced SSBs. Further, under these conditions, only the highest dose of X-ray in the presence of PpIX produced a few linear plasmids by inducing DSBs.

To confirm that PpIX enhanced X-ray irradiation-mediated DSBs, DNA ladder, as another kind of DNA molecule, and different concentrations of PpIX were mixed and irradiated with X-ray. Figure 3C shows an electropherogram of the X-ray-irradiated DNA ladder by capillary electrophoresis. The amplitudes of the DNA peaks were significantly decreased and showed a diffuse pattern following X-ray irradiation. Simple SSBs could not generate DNA fragments that show different molecular weights. In addition, nucleotide modifications and nucleotide replacements could not be detected under these conditions. These concepts suggest that the frequent SSBs that subsequently induce DSBs are responsible for our observations. For the same X-ray irradiation dose, a higher concentration of PpIX showed more SBs. Therefore, PpIX enhanced DSBs by X-ray irradiation.

### 2.4. PpIX Enhances Functional Decline of DNA by X-ray Irradiation

**Figure 4:**
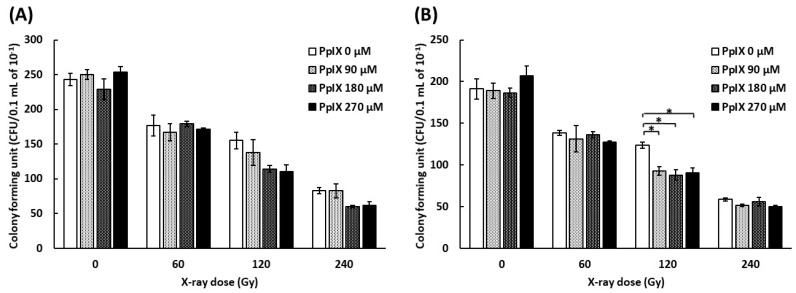
Evaluation of the functional decline of DNA by X-ray irradiation and enhancement by PpIX for plasmid pBR322. (**A**) The number of *E. coli* colony forming units (CFUs) on medium with ampicillin by transforming plasmid irradiated with X-ray at different concentrations of PpIX. (**B**) The number of *E. coli* colony forming units (CFUs) on medium with tetracycline. (Data given with *n* = 3, *: *p* < 0.05 in a one-way ANOVA and Tukey post-test)

To evaluate the function of plasmid, a colony formation assay was conducted by transforming *Escherichia coli* with plasmids mixed with PpIX and irradiated with X-ray. As pBR322 contains ampicillin and tetracycline resistance genes, we used media containing ampicillin or tetracycline. Figure 4 shows the number of colony-forming units (CFUs) by transforming plasmid mixed with PpIX and irradiated with X-ray. The number of CFUs decreased for every increase in X-ray dose. The number of CFUs decreased further as the PpIX concentration increased at 120 Gy and 240 Gy X-ray irradiation. ANOVA analysis did not show significant differences in the CFUs of plasmids irradiated with 120 Gy and 240 Gy on medium with ampicillin (Figure 4A) (*p* = 0.096 and 0.053, respectively). In contrast, ANOVA showed significant differences in the CFUs of plasmids irradiated with 120 Gy on medium with tetracycline (Figure 4B) (*p* = 0.004). Therefore, PpIX enhanced the functional decline of DNA by X-ray damage. The region of tetracycline repressor (TetR) was 1190 bp, and that of ampicillin (AmpR) was 860 bp. It was assumed that TetR was more likely to take damage than AmpR because TetR was longer than AmpR.

To evaluate the modification of the plasmid sequence, a next-generation sequencer read plasmid mixed with PpIX and irradiated with X-ray. Two regions of AmpR were targeted and analyzed. Alteration of the DNA sequence showed no significant difference across samples (Supplementary Materials Table S2).

### 2.5. PpIX Enhances Cellular Damage with DSBs by X-ray Irradiation

**Figure 5:**
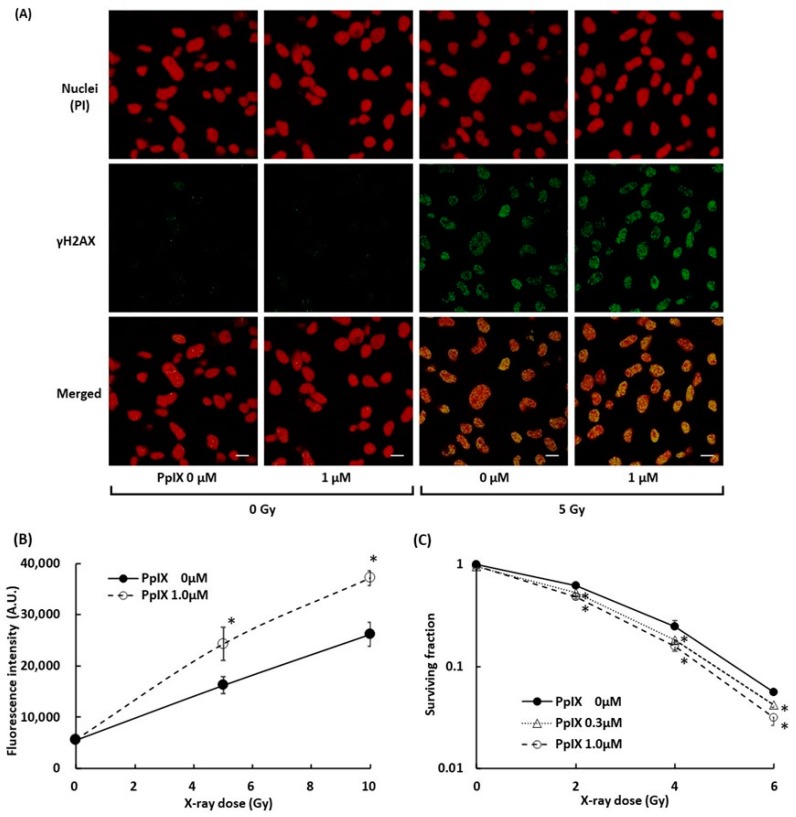
Evaluation of double-strand breaks (DSBs) within nuclei by X-ray irradiation and enhancement by PpIX. (**A**) Fluorescence in cell culture was imaged using laser confocal microscopy. Subcellular localization of γH2AX (green) and propidium iodide (PI)-stained nuclei (red) in cells with and without exposure to 1 µM PpIX or 5 Gy X-ray radiation. Scale bars: 20 µm. (**B**) The fluorescence intensity of γH2AX. (**C**) The WST-8 cell viability assay for cellular responses to PpIX treatment and X-ray irradiation. Data are the means ± SD (*n* = 4). Statistical significance (*p* < 0.01) relative to the experiment performed without PpIX at the same irradiation dose indicated by (*).

To evaluate the damage to B16-BL6 melanoma cells, we measured DSBs in the cell by γH2AX. γH2AX is known as a marker of DSBs within chromatin and to increase the relative intensity with an increase in the radiation dose [28,29]. Figure 5A displays fluorescence-labeled γH2AX in the nuclei of melanoma cells, and Figure 5B illustrates the signal intensity of fluorescence-labeled γH2AX. 5 Gy X-ray radiation induced γH2AX, and PpIX pre-incubation in combination with 5 Gy X-ray treatment further enhanced the γH2AX expression. These observations suggested that PpIX enhanced DSBs by X-ray irradiation. The WST-8 cell viability assay was performed to assess cell growth from PpIX treatment and X-ray irradiation. After seven days of irradiation treatment, the surviving fraction also decreased depending on PpIX concentration (Figure 5C).

## 3. Discussion

To understand the physicochemical reaction between X-ray and PpIX in RT, we first focused on ROS generation. We confirmed that PpIX increased the amount of ROS generated and the type of ROS identified (•OH and O_2_^•-^) by X-ray irradiation. •OH showed an increase regardless of the dissolved oxygen. Meanwhile, the increase in O_2_^•-^ was proportional to the amount of dissolved oxygen. This suggested that PpIX promoted the conversion of H_2_O to ^•^OH and also promoted conversion of oxygen to O_2_^•-^ by X-ray irradiation. The resistance of hypoxic cells to RT and chemotherapy is a major problem in the treatment of cancer [30,31]. Malignant melanoma is also considered radioresistant, and research is ongoing to improve the efficacy of therapy in hypoxia [32,33]. The PpIX property that enhances ^•^OH generation, which does not depend on the amount of dissolved oxygen, has the potential to improve RT effects of radioresistant tumors. Superoxide anion may involve the production of DNA SBs via metabolic process [34]. Therefore, the PpIX property that enhances O_2_^•-^ also has the potential to enhance RT effects.

Next, we focused on DNA SBs to understand the physicochemical reaction between X-ray and PpIX. We observed that X-ray irradiated plasmids showed a transition from supercoiled to relaxed forms. PpIX further enhanced the transition to relaxed forms, and linear forms were observed at the highest dose of X-ray (Figure 3). As reported previously, SSBs were confirmed by observation of the relaxed form of the plasmid [35]. This result suggested that both SSB and DSB generation was enhanced by the PpIX and X-ray irradiation combination. In this study, DSBs were not observed in the absence of PpIX on X-ray irradiation using a plasmid. It has been proposed that DSBs are generated by SSBs on the antisense strand close to the first SSB point [36,37,38]. Therefore, the experimental condition induced only SSBs, and the observation of DSBs might be explained by the aforementioned hypothesis. The functional depletion observed by X-ray exposure alone was further depleted by the combination of X-ray irradiation and PpIX. The sequence analysis of the plasmid treated with the combined treatment showed lower mutation frequency. These results suggested that plasmids were considerably damaged by the combination of X-ray and PpIX. Additionally, we found that X-ray-irradiated DNA ladders, as a kind of DNA molecule, were considerably broken down, as shown by capillary electrophoresis, differing from the plasmid. The amplitudes of the peaks were significantly reduced and showed a diffuse pattern. Although physicochemical verification using two types of DNA molecules required high concentrations of PpIX and X-ray irradiation, we confirmed that PpIX enhanced DNA SBs on X-ray exposure. DNA SBs are thought to cause apoptosis. Thus apoptosis is considered the main pathway for radiation-induced regulated cell death (RCD). In our previous in vivo study, we analyzed gene expression in tumor tissues after 5-ALA and X-ray treatment. X-ray treatment induced *Cdkn1a* (*p21*) and *Gadd45a*, and both genes were further induced following combined treatment with 5-ALA [6]. Pathological examinations revealed that fragmented nuclei were frequently observed in the 5-ALA and X-ray treatment groups [6]. From these results, we could determine that 5-ALA and X-ray treatment induced DSBs and apoptosis in tumor tissues. In addition, advances in molecular biology have revealed several pathways for radiation-induced RCD, including apoptosis, autophagy-dependent cell death, mitotic catastrophe, necrosis, and senescence-like cell death [39]. PpIX accumulation in cancer cells following X-Ray irradiation promotes ROS generation. ROS attacks not only DNA but also other biomolecules and could induce cancer cell death via a number of other pathways in addition to apoptosis, which is thought to be the trigger of the immune response.

A series of studies on the combination treatment of 5-ALA and ionizing radiation has been conducted by some research groups, and 5-ALA with subsequent intracellular PpIX accumulation has been found to increase kilo electron volt (KeV) or mega electron volt (MeV) irradiation cytotoxicity in a variety of contexts using mouse models of melanoma, glioma, colon cancer, etc. [5,6,7,8,9,10,11,12,13,14,15]. PpIX expression enhances ROS generation, which increases cellular DNA damage. However, the life span for ROS compounds is very short, especially those with an •OH, as this moiety has a very short half-life in vivo. Thus, DNA damage by •OH affects the intracellular localization of PpIX. On the other hand, PpIX is synthesized by exogenous 5-ALA in mitochondria through the heme synthesis pathway and diffuses into cells. Exogenous PpIX is lipophilic and taken up by cells with a diffuse cytoplasmic distribution, but it also exhibits tumor-selective accumulation. Therefore, the subcellular localization pattern differs between 5-ALA-induced endogenous- and exogenous PpIX [40]. This indicates that the effects of 5-ALA-induced PpIX and exogeneous PpIX are not the same. Additionally, exogenous PpIX causes phototoxicity, and 5-ALA has already been approved as a diagnostic drug for grade III and IV glioma in Europe, Japan, and the USA as a PpIX precursor [20]. For these reasons, 5-ALA, and not PpIX, is considered a radiosensitizer and is used in clinical practice and has been evaluated as a precursor in radiosensitized animal models. In this in vitro study, DSBs in melanoma cell nuclei were confirmed by γH2AX by X-ray irradiation and further enhanced by PpIX, as well as 5-ALA treatment in a previous study [8]. Further, the DSBs were accompanied by a decrease in cell surviving fraction, indicating that DNA damage enhanced by PpIX is one of the causes of anticancer activity. Thus, the dose of X-ray and PpIX or 5-ALA concentration, which causes cellular damage, could be applied to clinical treatment. The cancer cell-damaging mechanism is not only a physicochemical reaction but also a biological response.

5-ALA pre-treatment enhances the effectiveness of X-ray irradiation by acting as a radiomediator and facilitating PpIX accumulation in tumors, thereby enhancing ROS production, which subsequently further enhances DNA damage. These results suggest that 5-ALA and X-rays in combination (“radiodynamic therapy (RDT)”) instead of 5-ALA and laser beams (“photodynamic therapy (PDT)”) have the potential for treating RT. RDT may have additive effects for RT due to its involvement with the enhancement of •OH and O_2_^•-^ under hypoxia. Therefore, it could be employed to target radioresistant tumors, such as melanoma.

5-ALA is used as a precursor of PpIX for RDT. Due to differences in the enzymatic activity of the heme pathway between cancer and normal cells, cancer cells accumulate PpIX 10 to 20 times higher concentration than normal cells in the presence of 5-ALA [41]. To evaluate the side-effect of this treatment on the surrounding healthy tissues, we performed pathological examinations of the skin that interacts with the unattenuated X-rays [6]. Tumor-bearing C57BL/6J mice implanted with B16-BL6 melanoma cells were subsequently treated with irradiation (3 Gy/day for 10 days; total, 30 Gy) in addition to the local administration of 50 mg/kg 5-ALA 24 h prior to each irradiation treatment. Tumor growth was suppressed by X-ray irradiation and further suppressed following combined treatment with 5-ALA. Treatment with 5-ALA and X-ray irradiation produced mild parakeratotic, hyperkeratosis in H&E-stained skin sections, which were also exposed to 5-ALA. In contrast, treatment with X-ray alone yielded hyperplastic sebaceous glands associated with chronic inflammation. These results indicated that ALA-X-ray treatment might cause slight to moderate side-effects, but nothing significant. It was previously confirmed that the oral administration of 240 mg/kg 5-ALA 4 h prior to each irradiation (2 Gy/day for 30 days; total, 60 Gy) had caused no significant side effects on skin tissues (data not shown).

X-ray irradiation after treatment with PDT or Daylight PDT using 5-ALA has the same effect as RDT if PpIX is accumulated in the tumor. Since the peak concentration of PpIX is reached 4–6 h after 5-ALA administration, after which PpIX is metabolized and excreted from the tumor, it is necessary to time the X-ray irradiation while there is still PpIX in the tumor. There have been many studies on the combination of photochemotherapy using 5-ALA and RT, with the study by Berg *et al.* [42] being the first. However, these studies have examined the effects of PDT and X-rays and have not considered the physicochemical reaction of PpIX and X-rays. Like melanoma, nonmelanoma skin cancers, basal cell carcinoma (BCC), squamous cell carcinoma (SCC), and actinic keratoses (AKs) all exhibit some therapeutic response to 5-ALA PDT [43], and all are expected to experience a similar response when treated with X-ray radiation.

Recently, the advent of BRAF inhibitors, immune checkpoint inhibitors (ICIs), and their application as combination or sequential therapies have revolutionized clinical practice, leading to significant increases in survival rates for patients when compared to those for older cytotoxic treatment strategies for metastatic melanoma [44]. In addition, several retrospective analyses suggest that combining stereotactic radiosurgery (SRS) with active systemic therapies improves melanoma brain metastases control and prolongs survival without increasing toxicity. Preclinical and clinical data suggest that RT can cause disruption of the blood-brain barrier, enhancing drug delivery to the brain. Moreover, RT may increase the antitumor response by promoting antigen presentation and T-cell activation [45]. 5-ALA PDT can activate an otherwise quiescent local immune response under certain circumstances [46,47,48,49]. PDT may induce vascular shutdown by destroying endothelial cells and the vascular basement membrane, resulting in oxygen deprivation. Moreover, acute local inflammatory and immunological reactions, involving the innate and adaptive immune system, are induced as an indirect effect of PDT [46]. For RDT with 5-ALA, ionized calcium-binding adapter molecule 1 (Iba1)-positive macrophages were observed at the surface and within the subcutaneous tumors after treatment with multidose ionizing irradiation in combination with 5-ALA as demonstrated by the red immunohistochemical staining observed in samples from a rat glioma subcutaneous model [10]. Microarray analysis of tumor tissues following 5-ALA and X-ray treatment showed that GO terms, including ‘immune response’ and ‘defense response’, and genes related to MHC class I were slightly up-regulated in the X-ray treatment group and highly up-regulated in the 5-ALA and X-ray treatment group [6]. Thus, RDT with 5-ALA is expected to stimulate the immune system more than RT alone and has the potential to further our efforts to “harness synergistic biology between radiation and immunotherapy” [23].

## 4. Materials and Methods 

### 4.1. Materials

PpIX was purchased from MP Biomedicals (Illkirch, France). Aminophenyl fluorescein (APF) was purchased from Goryo Chemical (Sapporo, Japan). Dihydroethidium (DHE), dimethyl sulfoxide (DMSO), methanol, ethanol, PI, and PBS buffer were purchased from Wako Pure Chemical Industries Ltd. (Osaka, Japan). SOD was purchased from MP Biomedicals (Eschwege, Germany). φX174 Hae III digest and pBR322 (Accession number: GenBank J01749.1) were purchased from Takara Bio (Kusatsu, Japan). A DNA damage detection kit (containing γH2AX monoclonal antibody and secondary antibody) was purchased from Dojindo Laboratories (Kumamoto, Japan).

### 4.2. X-ray Irradiation

X-ray irradiation was carried out in a Faxitron CP-160 irradiator (Faxitron X-ray Corporation) with X-ray energy outputs of 160 kV. Evaluation of ROS measurement was performed at 1 Gy/min. Evaluation of reaction between PpIX and X-ray and resulting DNA breaks of DNA molecules was performed at 8 Gy/min.

### 4.3. Evaluation of ROS Production

To measure hydroxyl radical (^•^OH) generation, APF was used as a detection agent [50]. APF fluorescence was measured using a plate reader (infinite M200, TECAN, Kawasaki, Japan) at Em: 490 nm and Ex: 515 nm. The reaction mixture was prepared in a final volume of 100 µL in a 96-well black plate with the following reagents at indicated final concentrations: 5 µM PpIX, 5 µM APF, 0.25% DMSO, and PBS buffer. Ethanol (25%) was added to scavenge ^•^OH radicals. The reaction mixture was irradiated with 5 Gy and 10 Gy, and the fluorescence was measured. To measure superoxide radical (O_2_^•-^), DHE was used as a detection agent [51]. DHE fluorescence was measured using a microplate reader at Em: 485 nm and Ex: 610 nm. The reaction mixture was prepared as mentioned above (for the measurement of ^•^OH), except for replacing 5 µM APF with 50 µM DHE. Superoxide dismutase (30 U/mL) was added to scavenge O_2_^•-^. The reaction mixture was irradiated with 5 Gy and 10 Gy, and the fluorescence was measured.

To evaluate the effect of dissolved oxygen on ROS generation, the reaction mixture was bubbled with N_2_, air, or O_2_ gas. PpIX (5 µM, 500 µL) and 5 µM APF or 50 µM DHE were placed in airtight 5 mL containers with gas inlet and outlet ports. After containers were bubbled with N_2_, air, or O_2_ gas for 2 min, the inlet and outlet valves were closed, and the containers were immediately irradiated with X-ray. After X-ray irradiation, the fluorescence of each mixture was measured.

### 4.4. Evaluation of DNA Break

To confirm that PpIX enhances DNA breaks induced by X-ray irradiation, liner and circular double-stranded DNA were employed. For the detection of liner DNA breaks, DNA ladder φX174 Hae III digest (50 ng/µL) was added to 0, 90, and 270 µM PpIX. Each mixture was irradiated with 60 Gy, 120 Gy, and 240 Gy. Irradiated DNA ladder was analyzed by capillary gel electrophoresis (Agilent 2100 bioanalyzer, Agilent, Santa Clara, CA, USA) with Agilent DNA 12,000 kit (Agilent, Santa Clara, CA, USA).

To evaluate circular DNA breaks, pBR322 plasmid (50 ng/µL) was added to 0, 90, 180, and 270 µM PpIX. Each mixture was irradiated with 60 Gy, 120 Gy, and 240 Gy. The irradiated plasmid was analyzed by agarose gel electrophoresis, and fluorescence intensities of plasmid bands were quantified using NIH Image software ImageJ (http://rsb.info.nih.gov/ij/).

### 4.5. Evaluation of DNA Function by Colony Formation Assay

To evaluate the function of plasmid, colony formation assay was performed by transforming *Escherichia coli* with irradiated pBR322. A mixture of 1 µL of 50 ng/µL irradiated pBR322 and PpIX was added into 20 µL competent *E. coli* K12 JM109 with genotype *rec*A1, *end*A1, *gyr*A96, *thi-*1, *hsd*R17*(r_k_^-^*, *m_k_^+^)*, *e*14^-^ *(mcr*A^-^*)*, *sup*E44, *rel*A1, Δ*(lac-pro*AB*)/*[F′ *tra*D36, *pro*AB^+^, *lac*I^q^, *lac*ZΔM15] and incubated on ice for 30 min. After heat shock at 42 °C for 1–2 min, 100 µL of SOC medium (2% vegetable peptone, 0.5% yeast extract, 10 mM NaCl, 2.5 mM KCl, 10 mM MgCl_2_, 10 mM MgSO_4_, 20 mM glucose) was added and incubated at 37 °C for 30 min. The cell suspension was diluted 10-fold, and 100 µL was plated on Luria Bertani Agar medium (1% tryptone, 0.5% yeast extract, 1% NaCl, 1.5% agarose) supplemented with 20 µg/mL ampicillin or tetracycline. Agar medium was incubated at 37ºC for 16 h, and colonies formed were counted.

### 4.6. Cell Culture

B16-BL6 mouse melanoma cell line was supplied by Riken Cell Bank (Tsukuba, Japan). Cells were cultured in RPMI1640 (Fujifilm Wako Pure Chemical Corporation, Osaka, Japan) containing 10% FBS in a 5% CO_2_ humidified incubator at 37 °C. The medium was supplemented with 100 units/mL penicillin and 100 μg/mL streptomycin.

### 4.7. γH2AX Detection

To observe the distribution of γH2AX, cells were separately grown on glass chamber slides. PpIX was added 4 h before X-ray irradiation in culture medium at 1 μM. Control cells were incubated without PpIX. After the medium was replaced with fresh culture medium, cells were irradiated by an X-ray irradiator. The cells were fixed 30 min after irradiation. The cells were stained with γH2AX using a secondary antibody tagged with green fluorescence, while DNA was counter-stained with propidium iodide (PI) (0.5 μg/mL). Cells were treated with RNase (0.25 mg/mL) prior to staining with PI. Cells were finally imaged using a laser confocal microscope (Nikon AI Confocal Scanning Laser Microscope, Nikon Instruments Inc., Tokyo, Japan), with a 60 X oil-immersion objective lens. For the measurement of γH2AX using a flow cytometer, B16 cells were cultured in 25 cm^2^ flask until they became confluent, and radiated by X-rays. The cells were collected and fixed 30 min after irradiation. The cells were stained with anti-γH2AX antibody using a secondary antibody tagged with green fluorescence. The cells were then analyzed using a FACSCalibur flow cytometer (BD Biosciences, San Diego, CA, USA).

### 4.8. Cell Viability Assay

The WST-8 cell viability assay was performed to assess cellular responses to PpIX treatment and X-ray irradiation. After exposure to the doses of 0 to 6 Gy of X-ray radiation, the cells were seeded in 96-well plates with a seeding density of 50 cells/well and incubated at 37 °C and 5% CO_2_. After seven days of irradiation treatment, WST-8 assays were performed. The plates were incubated with fresh medium containing 10% WST-8 in a 5% CO_2_ humidified incubator at 37 °C for 1 h, followed by measurement of the absorbance at 450 nm against a referenced absorbance at 600 nm using a plate reader. Relative cell viability was defined as the dye absorption ratio of treated versus untreated cells.

### 4.9. Statistical Analysis

Statistically significant differences in each X-ray dose condition were determined using a one-way analysis of variance (ANOVA) followed by Tukey’s posthoc analysis. A *p*-value of <0.05 was considered statistically significant. Data are presented as mean ± standard error of the mean.

## 5. Conclusions

This study was conducted to elucidate the anticancer mechanism of 5-ALA-induced PpIX and X-ray irradiation for mainly physics-based evaluation. Two kinds of ROS generations, •OH and O_2_^•-^, by the interaction between PpIX and X-ray were identified, and •OH showed an increase regardless of the dissolved oxygen. Meanwhile, the increase in O_2_^•-^ was proportional to the dissolved oxygen. SBs of DNA molecule were evaluated by gel electrophoresis. SBs of two types of DNA molecule, plasmid as a coiled DNA and DNA ladder as a linear DNA, were analyzed. We found that PpIX enhanced both SSBs and DSBs under X-ray irradiation. It was confirmed that PpIX further enhanced SBs compared to X-rays alone. It was also confirmed that these reactions occurred in melanoma cell nuclei.

5-ALA, as a precursor to PpIX, has been approved for use in PDD and can be administered orally. It is a great advantage for RT performed with fractionated irradiation. Since PpIX can enhance ROS generation even in a hypoxic state and induce DNA damage, 5-ALA RDT is expected to improve therapeutic efficacy for radioresistant malignant melanoma.

## Supplementary Materials

The following are available online at https://www.mdpi.com/1422-0067/21/7/2302/s1, Evaluation of the integrity of DNA by sequencing.

## Abbreviations

5-ALA	5-aminolevulinic acid
APF	3′-(p-aminophenyl) fluorescein
DHE	dihydroethidium
DSB	double-strand break
PDD	photodynamic diagnosis
PDT	photodynamic therapy
PpIX	protoporphyrin IX
RDT	radiodynamic therapy
ROS	reactive oxygen species
RT	radiotherapy
SB	strand break
SSB	single-strand break
