# Supplementary material for: DNA Strand Break Properties of Protoporphyrin IX by X-ray Irradiation against Melanoma
Source: Int J Mol Sci. 2020 Mar 26;21(7):2302. doi: 10.3390/ijms21072302 (PMC7177738; doi:10.3390/ijms21072302)
Supplement: Supplementary file 1 [file ijms-21-02302-s001.pdf]

Supplementary material

Evaluation of integrity of DNA by sequencing

To measure integrity of plasmid, plasmid sequence was read by Next Generation Sequencing (NGS). Two ampicillin resistance ($Amp^r$) regions on pBR322 were amplified by polymerase chain reaction (PCR) (Taq 2x Master Mix, BioLabs, MA, USA) using respective primer pairs (Table 1). Samples were submitted to the Gifu University Next Generation Sequencing service, which employs Illumina MiSeq. Sequence data were subjected to quality filtering with a quality score threshold of 30. Mutation rates were evaluated by NGS-eval (http://www.ibi.vu.nl/programs/ngsevalwww/).

Table 2 shows alteration of DNA sequence. Alteration of DNA sequence showed no significant difference across samples.

**Table S1.** Primers for NGS. Underlined parts represent target sequences for $Amp^r$ region of pBR322. The remaining parts represent sequences analyzed by Miseq.

| Target | Sequence |
|---|---|
| 3313-3767 | 5′ - TCGTCGGCAGCGTCAGATGTGTATAAGAGACAG-<u>AGGCACCTATCTCAGCG</u>-3′ |
| | 5′ -GTCTCGTGGGCTCGGAGATGTGTATAAGAGACAG-<u>TAACACTGCGGCCAACT</u>-3′ |
| 3660-4135 | 5′ - TCGTCGGCAGCGTCAGATGTGTATAAGAGACAG–<u>GGTTCCCAACGATCAAG</u>-3′ |
| | 5′ -GTCTCGTGGGCTCGGAGATGTGTATAAGAGACAG-<u>CGTGTCGCCCTTATTCC</u>-3′ |

**Table S2.** Modification of DNA sequence on two regions of $Amp^r$. $Amp^r$ region spans nucleotides 3293 to 4135.

| region | X-ray (Gy) | PpIX (μM) | Modification (%) |
|---|---|---|---|
| **3313-3767** | **0** | **0** | **0.26** |
| | **0** | **270** | **0.26** |
| | **240** | **0** | **0.25** |
| | **240** | **270** | **0.27** |
| **3660-4135** | **0** | **0** | **0.38** |
| | **0** | **270** | **0.51** |
| | **240** | **0** | **0.38** |
| | **240** | **270** | **0.28** |
